# ELDGG: an end-to-end LiDAR-dynamic-guided GAN for hyperspectral image hierarchical reconstruction and classification

**DOI:** 10.1038/s41598-025-30660-8

**Published:** 2025-12-15

**Authors:** Xingyue Zhang, Mingju Chen, Senyuan Li, Xiao Hu, Zhengxu Duan, Yangming Luo, Chen Xie, Xingzhong Xiong

**Affiliations:** 1 School of Automation and Information Engineering, Sichuan University of Science & Engineering, Yibin, 644002 China; 2 Intelligent Perception and Control Key Laboratory of Sichuan Province, Sichuan University of Science & Engineering, Yibin, 644002 China; 3 Yibin Research Institute of University of Electronic Science and Technology of China, Yibin, 644002 China; 4 Sichuan University of Arts and Science, Dazhou, 635002 China

**Keywords:** HSI-LiDAR fusion, Land cover classification, Generative adversarial network, Implicit neural representation, Deep learning, Engineering, Mathematics and computing, Optics and photonics

## Abstract

To address the prevalent issues in the classification of hyperspectral image (HSI) and light detection and ranging (LiDAR) data fusion, such as insufficient dynamic adaptive interaction of cross-modal features, and difficulties in high-fidelity spatial detail reconstruction, this paper proposes an end-to-end LiDAR-dynamic-guided GAN for hyperspectral image hierarchical reconstruction and classification (ELDGG). The core framework of the network consists of a guided hierarchical reconstruction generator (GHR-Generator) and a perception-enhanced spectral regularization discriminator (PSR-Discriminator). First, we propose the cross-modal parameter-adaptive fusion module (CPAF-Module), which leverages the global context of LiDAR data to generate dynamic convolutional operators tailored for HSI features, addressing the limitations of static fusion methods. Second, to enhance the reconstruction quality of spatial details, we design the LiDAR-guided neural implicit field reconstruction unit (L-GNIF Unit). By learning a continuous mapping from coordinates to features, it achieves high-fidelity and artifact-free feature space reconstruction. Furthermore, we innovatively integrate spectral normalization constraints with a multi-level feature matching mechanism to construct the PSR-Discriminator. This discriminator provides more comprehensive perceptual signals across three scales: shallow textures, mid-level structures, and deep semantics. The entire framework is optimized through end-to-end training and a joint multi-task optimization loss function, ensuring that the generated fused features exhibit both authenticity and class discriminability. On this basis, we further design a spatial-spectral refinement classifier (SSR-Classifier) to accurately decode the deeply optimized feature maps, ultimately producing high-precision land cover classification results. Experiments demonstrate ELDGG’s superiority over state-of-the-art methods in both fusion quality and classification accuracy.

## Introduction

Hyperspectral imaging achieves fine-grained classification by capturing ground object reflectance spectra, generating hyperspectral image (HSI) with hundreds or even thousands of continuous spectral bands that precisely characterize spectral features of targets^1^. However, HSI data face several key challenges, including information redundancy caused by spatial, spectral, and spatial-spectral correlations between bands^2^, the lack of three-dimensional structural information due to its inherently two-dimensional planar nature^3^, and the “same object with different spectra” or “different objects with similar spectra” issues resulting from signal attenuation under adverse weather conditions^4^.

To address these limitations, researchers have increasingly explored the fusion of HSI with other modal information to achieve more stable and generalized feature representation. For instance, the LDBMamba model^5^ enables cross-domain generalization of hyperspectral images through a language-guided mechanism, demonstrating the potential of textual modality in enhancing the semantic-level understanding of spectral features. The LG-GCN model^6^ achieves collaborative modeling of multi-scale spectral features. Furthermore, hyperspectral data can be complementarily fused with modalities such as panchromatic (PAN), multispectral (MSI), light detection and ranging (LiDAR), and synthetic aperture radar (SAR), leading to significant performance improvements in tasks such as spatial resolution enhancement, structural feature extraction, and scene understanding.

Among the various fusion-capable modalities, LiDAR stands out as one of the most representative paired data sources due to its high complementarity with hyperspectral imagery in terms of detection mechanisms. LiDAR rapidly acquires high-precision 3D spatial information through laser pulses, excelling particularly in vertical structure detection. It is also less susceptible to weather and lighting interference, enabling stable acquisition of geometric and elevation features of ground objects^7^. However, LiDAR data lacks spectral attribute information, making it difficult to distinguish between objects with similar geometries but different material properties^8^. Therefore, the fusion of hyperspectral and LiDAR data not only compensates for the limitations of individual modalities but also promotes the transition of remote sensing technology from two-dimensional monitoring to a “spatial + spectral + structural + temporal” four-dimensional dynamic analysis paradigm. This integrated approach holds significant application potential in fields such as climate change research, ecological monitoring, urban expansion analysis, and precision agriculture^9,10^.

Among various applications, achieving high-precision land cover classification is a core task for unlocking the value of HSI-LiDAR fusion classification, making classification-oriented fusion methods a key research focus. Current fusion classification approaches can be broadly categorized into traditional methods and deep learning (DL)-based methods. Traditional techniques rely on feature engineering and machine learning, extracting spatial-spectral-elevation features using morphological profiles (MP)^11^, attribute profiles (AP), and extinction profiles (EP), combined with support vector machines (SVM) for classification^12^. Representative methods include AP-based fusion framework^13^and ILCP^14^, which achieved notable results but remain limited by manual feature design, particularly in complex scenes and shadowed areas. DL, with its powerful automatic feature extraction, nonlinear modeling, and end-to-end optimization, has revolutionized HSI-LiDAR fusion classification^15^. Convolutional neural network (CNN) architectures, such as three-branch CNN^16^ and AFC-CNN^17^, effectively integrate multi-source data features through diverse strategies. Recently, generative adversarial networks (GAN) and architectures such as Transformer have further advanced the development of multimodal representation learning. For example, MLIF-AL network^18^ and D3Net^19^ have achieved excellent performance in spectral-structural feature interaction by leveraging adversarial mechanisms. Building upon this, the introduction of the Mamba architecture has provided a new modeling paradigm for hyperspectral-LiDAR fusion. While retaining the powerful modeling capabilities of Transformer, this architecture significantly improves computational efficiency and robustness in sequence modeling. For instance, the E-Mamba^20^ enhances information coupling through dynamic transmission of cross-modal features, while the AMSFN^21^ achieves efficient feature integration via a sequence-driven fusion mechanism, outperforming current mainstream CNN, Transformer, and Mamba models in both classification accuracy and computational efficiency. Additionally, emerging techniques like contrastive learning^22^ and geometric algebra^23^ provide new avenues for enhancing model interpretability and generalization.

Despite the emergence of numerous high-performance models and significant progress in deep learning-based HSI-LiDAR fusion classification methods, current approaches still face three critical bottlenecks: the limitations of static feature fusion, distortion in spatial detail reconstruction and physical authenticity of features. The existence of these problems restricts the performance ceiling of the collaborative classification of hyperspectral and LiDAR data.

In response to these challenges, this study develops the end-to-end LiDAR-dynamic-guided GAN for hyperspectral image hierarchical reconstruction and classification (ELDGG), aiming to achieve deeper and higher-fidelity collaborative classification between HSI and LiDAR data. Our principal contributions are threefold:

1. To address the limitations of static strategies commonly employed in the fusion stage by conventional methods, we designed the cross-modal parameter adaptive fusion module (CPAF-Module). This module achieves parameter-level dynamic fusion, capable of adaptively adjusting the fusion strategy according to the characteristics of the input data. This enables our model to more intelligently handle complex ground object scenarios, effectively enhancing both the fusion performance and subsequent classification accuracy.
2. To overcome common issues in the feature reconstruction process, such as spatial detail distortion and artifacts, we designed the LiDAR-guided neural implicit field reconstruction unit (L-GNIF Unit). This unit utilizes the precise geometric information provided by LiDAR data as a strong guiding signal for reconstructing spatial features, modeling and reconstructing spatial characteristics in the form of continuous functions. This enables high-fidelity reconstruction of ground object features, demonstrating exceptional performance particularly in preserving critical details such as object boundaries.
3. To tackle the potential lack of physical realism in features, we constructed a generative adversarial network incorporating a perception-enhanced spectral regularization discriminator (PSR-Discriminator). Through a carefully designed joint multi-task loss function, it simultaneously constrains feature distribution consistency and physical realism. This approach not only improves the credibility of classification results but also significantly enhances the model’s generalization capability in unseen scenarios.

## Theoretical methods

**Fig. 1:**
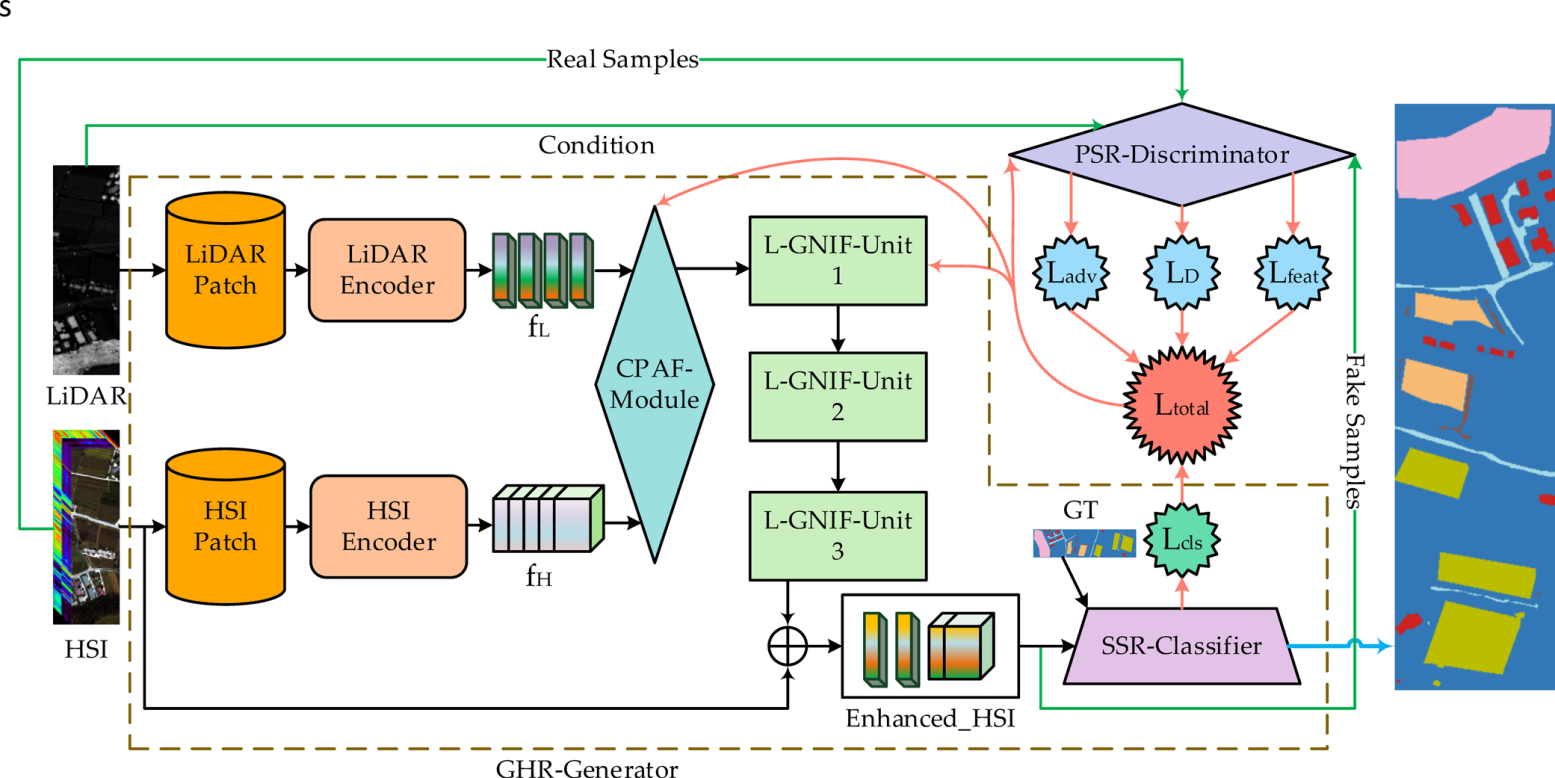
ELDGG framework structure diagram.

The proposed ELDGG framework in this paper is shown in Fig. 1, which consists of two core networks: the guided hierarchical reconstruction generator (GHR-Generator) and the PSR-Discriminator. The following subsections will provide a detailed analysis of each core component.

### GHR-generator

The GHR-Generator adopts an end-to-end architecture design, achieving complete processing from multi-modal data input to land cover classification output. It consists of preprocessing and encoding, CPAF-Module, L-GNIF Unit, and SSR-Classifier.

#### Preprocessing and encoding

To transform the original $HSI \in {\mathbb{R}}^{{C_{{{\text{hsi}}}} \times H_{hsi} \times W_{hsi} }}$ and $LiDAR \in {\mathbb{R}}^{{C_{lidar} \times H_{lidar} \times W_{lidar} }}$ into information-rich and standardized multi-modal data blocks suitable for deep model processing, this paper first performs systematic preprocessing on the raw data. Specifically, it includes: reflective boundary padding to alleviate the problem of missing edge information in images, combined with a sliding window strategy to extract spatially context-aware data blocks (with size P × P), and normalization processing with data augmentation operations to improve the model’s training stability and generalization capability.

Building upon this, to further extract deep-level feature information from the two modalities, the preprocessed paired $HSI \in {\mathbb{R}}^{{C_{{{\text{hsi}}}} \times P \times P}}$ and $LiDAR \in {\mathbb{R}}^{{C_{lidar} \times P \times P}}$ patches are fed into a dual-stream convolutional encoder architecture for parallel feature extraction. This dual-stream structure consists of an HSI encoder and a LiDAR encoder, which share identical network configurations but maintain independent parameters. The HSI encoder primarily focuses on capturing spectral-spatial joint features, enhancing the modeling of inter-band correlations, while the LiDAR encoder specializes in extracting elevation and geometric structure features, providing spatial structural priors for subsequent modality-guided fusion. Each encoder comprises two convolutional blocks: the first layer employs 3 × 3 convolutions to extract shallow spatial features, and the second layer performs feature downsampling via stride-2 convolutions while preserving local context, thereby extracting more semantic-rich high-level representations. Each convolutional layer is followed by batch normalization and a LeakyReLU (ReLU) activation function to accelerate convergence and mitigate gradient vanishing. The resulting two sets of deep feature maps,$f_{H} \in {\mathbb{R}}^{C \times P \times P}$ and $f_{L} \in {\mathbb{R}}^{C \times P \times P}$, serve as inputs to the subsequent multimodal fusion module. Through this dual-stream parallel design, the model achieves feature decoupling and semantic alignment between modalities at an early stage, providing subsequent modules with rich and complementary deep representations, thereby laying a solid foundation for high-accuracy land cover classification.

#### CPAF-module

In hyperspectral-LiDAR fusion, existing methods mostly adopt simple concatenation or element-wise addition approaches, neglecting the deep-level contextual relationships between modalities. Some studies have attempted to introduce attention mechanisms to perform weighted adjustment of existing features through learned weights. Although this improves fusion performance to some extent, it essentially remains a static linear fusion approach, lacking more flexible and expressive modality interaction mechanisms. To address this, we propose a parameter-level adaptive fusion strategy that enables one modality (LiDAR) to directly participate in and guide the feature processing of another modality (HSI). Based on this strategy, we construct the cross-modal parameter adaptive fusion module (CPAF-Module), which fully utilizes the global spatial structural information contained in LiDAR to dynamically generate dedicated convolution operators for HSI features, achieving a transition from “fixed processing” to “modality-aware processing”.

**Fig. 2:**
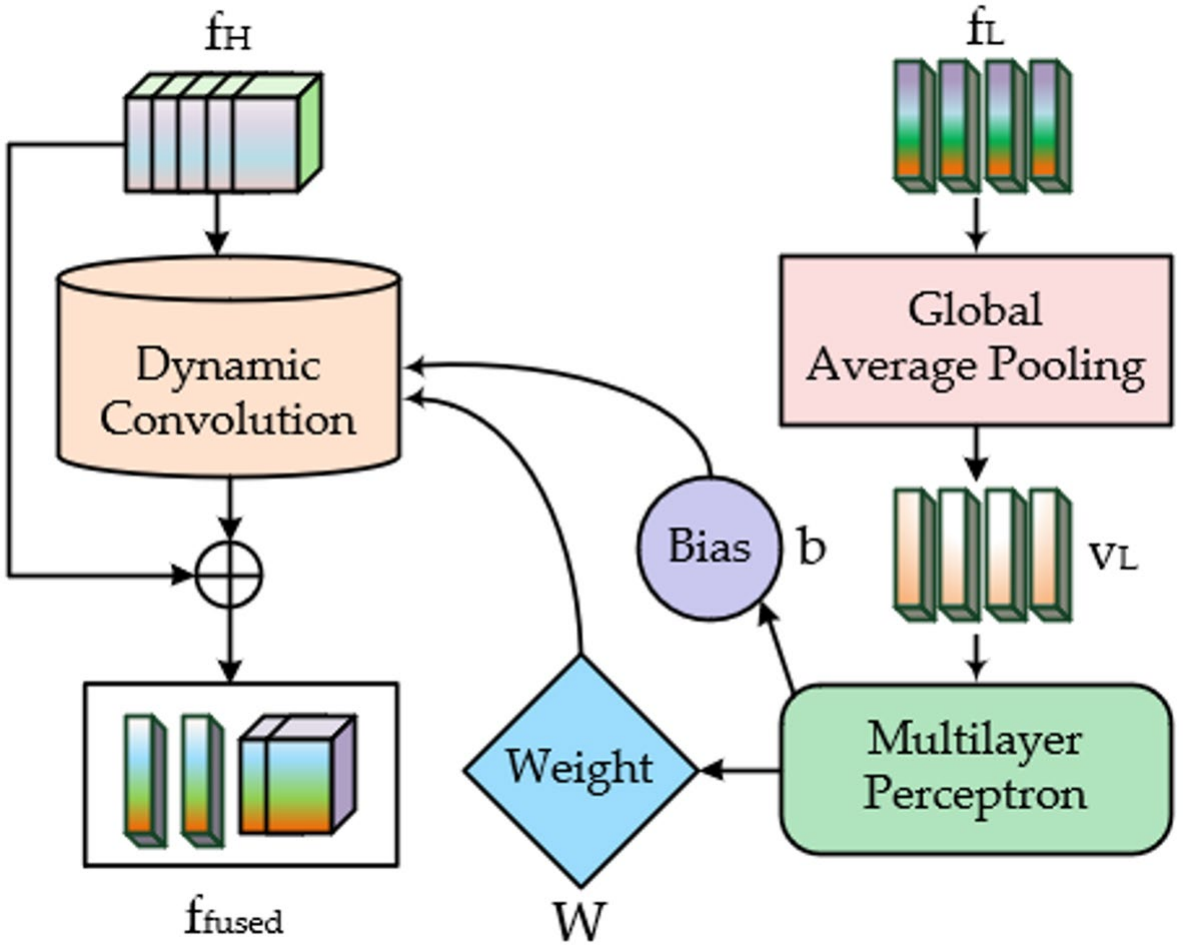
The structure diagram of the CPAF-Module.

Unlike traditional convolutional networks that use fixed kernels, the CPAF-Module automatically generates adaptive convolution kernel parameters based on input LiDAR features, providing differentiated processing mechanisms for each input sample. For example, in flat terrain regions represented by LiDAR, the generated convolution kernels may favor smoothing operations, while in areas with building edges or abrupt object changes, the kernels emphasize edge preservation and structural response. The structure of this module is shown in Fig. 2, and its complete implementation consists of the following three key steps:

1. Global spatial context extraction:

First, global average pooling is applied to the preprocessed LiDAR feature map $f_{L}$ to capture its rich spatial structural information, compressing it into a high-dimensional context vector $v_{L}$. This vector effectively characterizes the overall spatial distribution features of the current input sample, with the specific expression as follows:

1 $$v_{L} = GlobalAvgPool\left( {f_{L} } \right)$$

(2) Dynamic convolution parameter generation:

Subsequently, this context vector $v_{L}$ is fed into a lightweight parameter generator $\phi$ to dynamically generate the weight parameters W and bias terms b required for the HSI feature convolution operation. This parameter generator $\phi$ adopts a multi-layer perceptron (MLP), architecture consisting of two fully connected layers: the first layer maps $Linear_{1}$ the LiDAR context vector from dimension $C$ to a 256-dimensional latent space, with ReLU activation applied to enhance nonlinear representation capability. In the second layer $Linear_{2}$, to control parameter volume while achieving position-agnostic dynamic adaptation, we employ a 1 × 1 convolutional kernel structure to further project the latent vector into convolutional parameter sets. The output dimensions are designed to match the HSI feature channels $C$, producing both the weight matrix $W \in {\mathbb{R}}^{C \times C}$ and bias vector $b \in {\mathbb{R}}^{C}$. The specific procedure can be formulated as follows:

2 $$\left( {W,b} \right) = \phi \left( {v_{L} } \right) = Linear_{2} \left( {{\text{Re}} LU\left( {Linear_{1} \left( {v_{L} } \right)} \right)} \right)$$

Through this mechanism, LiDAR features from different regions can guide the network to apply differentiated convolutional processing in the HSI space, enhancing structural alignment between modalities.

(3) Guided fusion with residual enhancement:

Finally, the dynamically generated parameters are used to perform convolution on the HSI feature map $f_{H}$, yielding structurally guided features $f_{fused}$. Simultaneously, a residual connection mechanism is introduced to preserve the original HSI information, thereby obtaining deeper and more structurally flexible multi-modal representations after fusion:

3 $$f_{{f_{used} }} = f_{H} + Conv2D\left( {f_{H} ,\ker nel = W,bias = b} \right)$$

#### L-GNIF unit

**Fig. 3:**
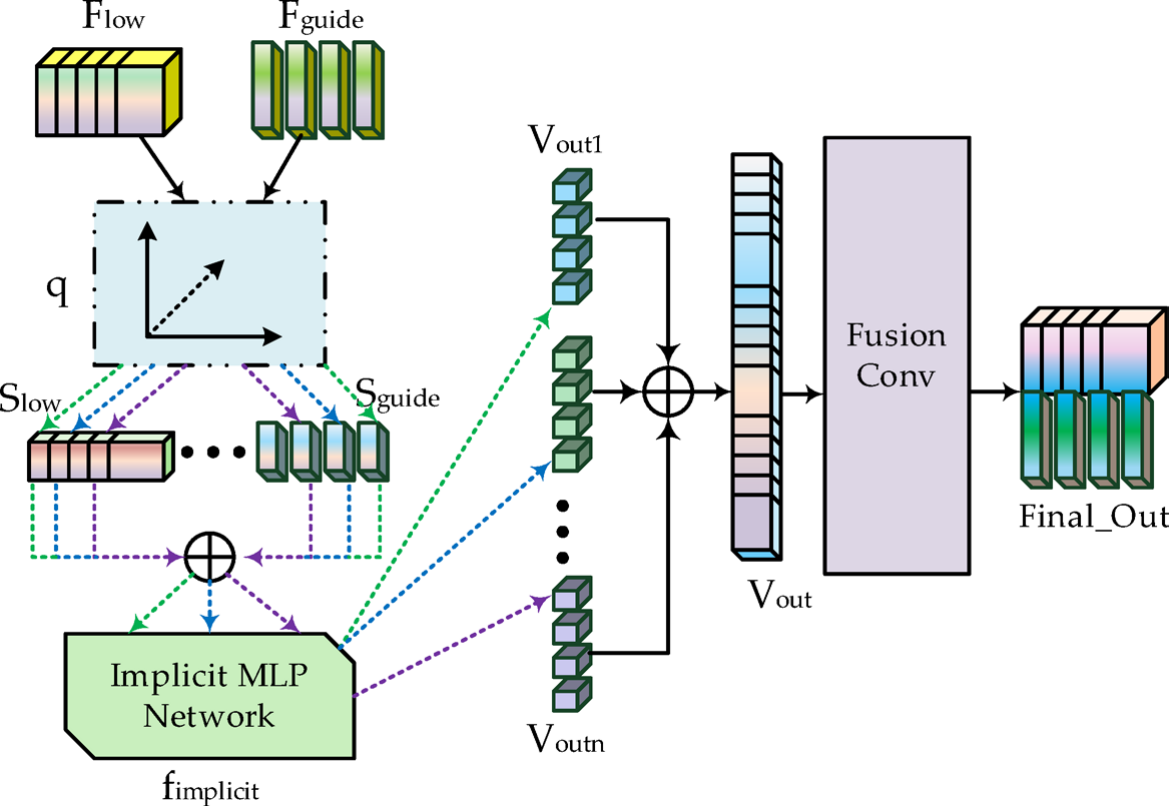
The structure diagram of L-GNIF Unit.

In deep convolutional networks, feature maps undergo progressive down-sampling through the encoder to extract high-level semantic information. However, this process often leads to the loss of spatial information, particularly during subsequent decoding and reconstruction stages. Effectively recovering these lost structural details becomes a critical challenge. Conventional up-sampling methods suffer from inherent issues such as blurred edges and textures, loss of high-frequency information, and checkerboard artifacts, which significantly compromise downstream land cover parsing accuracy. To address these limitations, we propose the L-GNIF Unit, whose architecture is illustrated in Fig. 3. This module adopts the implicit neural representation (INR) paradigm to construct a continuous function $f_{implicit}$ that can be evaluated at any spatial coordinate $q = \left( {x,y} \right)$, combining low-resolution features with high-resolution guidance features from LiDAR to precisely predict high-quality feature representations at arbitrary locations. Its core formulation is as follows:

4 $$v_{out} = f_{implicit} \left( {concat\left( {F_{low} \left( q \right),F_{guide} \left( q \right),q} \right)} \right)$$

Here,$F_{low\left( q \right)}$ represents the feature vector obtained through bilinear interpolation on low-resolution feature maps at coordinate $q$, while $F_{guide} \left( q \right)$ denotes guidance features sampled from high-resolution LiDAR feature maps-the key component ensuring reconstructed details align with actual spatial structures. The normalized coordinate $q$ within [0,1] range provides spatial awareness to the network. Through this continuous modeling approach, the network effectively reduces artifacts and blurring without relying on discrete interpolation operations, while demonstrating outstanding performance in high-frequency information recovery and edge preservation.

**Fig. 4:**
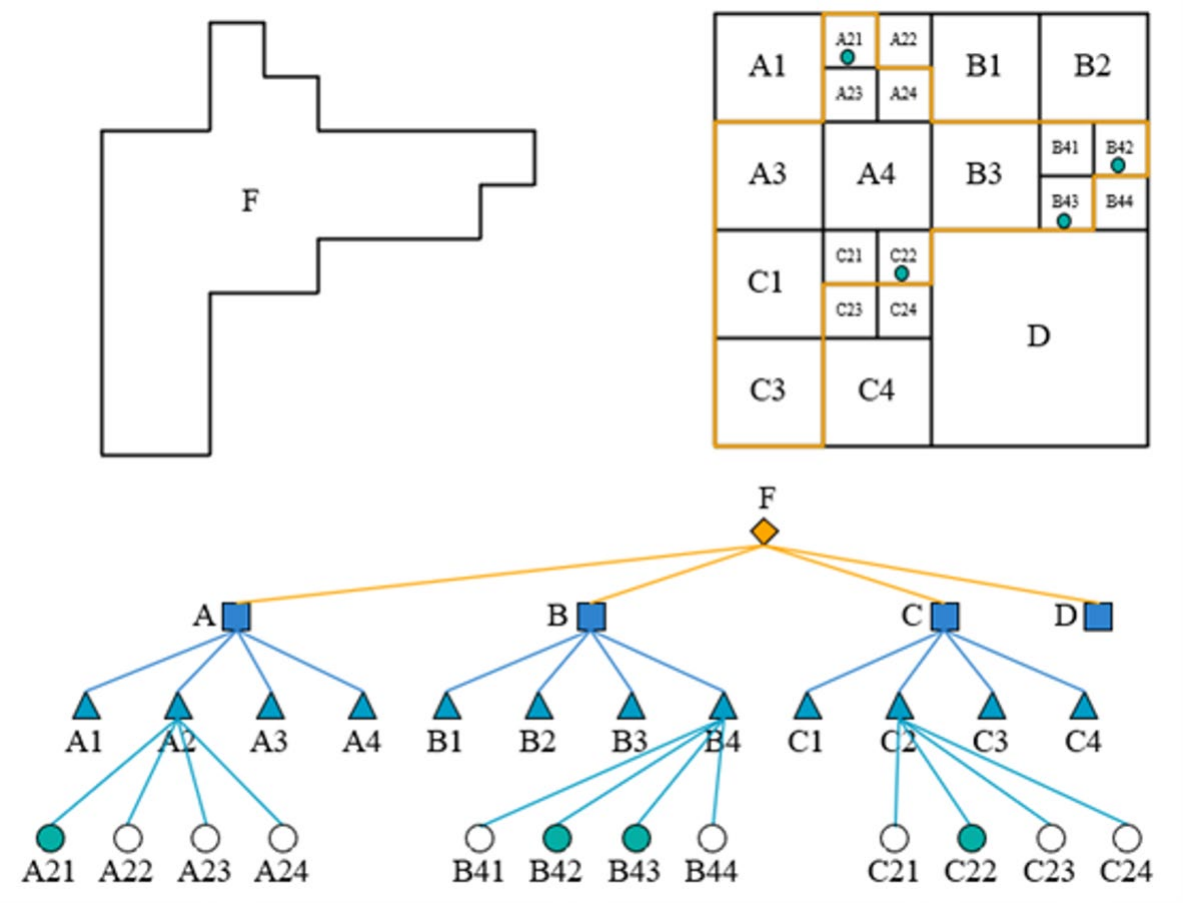
The structure diagram of a quadtree.

Building upon this foundation, to achieve effective recovery from deeply compressed features to high-resolution outputs, we introduce a quadtree-inspired hierarchical reconstruction architecture. This architecture draws inspiration from the top-down refinement concept of spatial decomposition in quadtrees^24^ (see Fig. 4 for structure illustration), this architecture cascades three L-GNIF Units, each operating at a different resolution, to progressively reconstruct feature maps from “coarse contours” to “fine-grained details.” Within this hierarchical system, each L-GNIF Unit functions as a flexible refinement module operating at a specific scale, rather than directly and rigidly corresponding to a single quadtree node. Information transfer and integration between different levels are achieved through recursive residual fusion: the unit at level 1 receives the reconstructed features $F_{rec}^{{\left( {l - 1} \right)}}$ from the previous level along with the aligned LiDAR guidance features $F_{guide}^{\left( l \right)}$, generating the reconstruction output for the current level:

5 $$F_{rec}^{\left( l \right)} = f_{implicit}^{\left( l \right)} \left( {F_{rec}^{{\left( {l - 1} \right)}} ,F_{guide}^{\left( l \right)} ,q^{\left( l \right)} } \right),l = 1,2,3$$

Specifically, the first level takes the fused features $f_{fused}$ as input, combined with × 2 up-sampled LiDAR guidance features, to reconstruct coarse-grained global contours $F_{rec}^{\left( 1 \right)}$; the second level processes $F_{rec}^{\left( 1 \right)}$ with × 3 up-sampled guidance to enhance medium-scale boundaries and textures $F_{rec}^{\left( 2 \right)}$; the third level refines high-frequency details $F_{rec}^{\left( 3 \right)}$ based on $F_{rec}^{\left( 2 \right)}$ using × 4 up-sampled guidance. Finally, the output $F_{rec}^{\left( 3 \right)}$ from the third level is restored to the original size P × P through a 1 × 1 convolution and adaptive pooling operation, and fused with the original HSI input via residual connection:

6 $$X_{hsi}^{final} = X_{hsi}^{original} + AdaptivePool_{P \times P} \left( {Conv_{1 \times 1} \left( {F_{rec}^{\left( 3 \right)} } \right)} \right)$$

#### Spatio-spectral refinement classifier

**Fig. 5:**
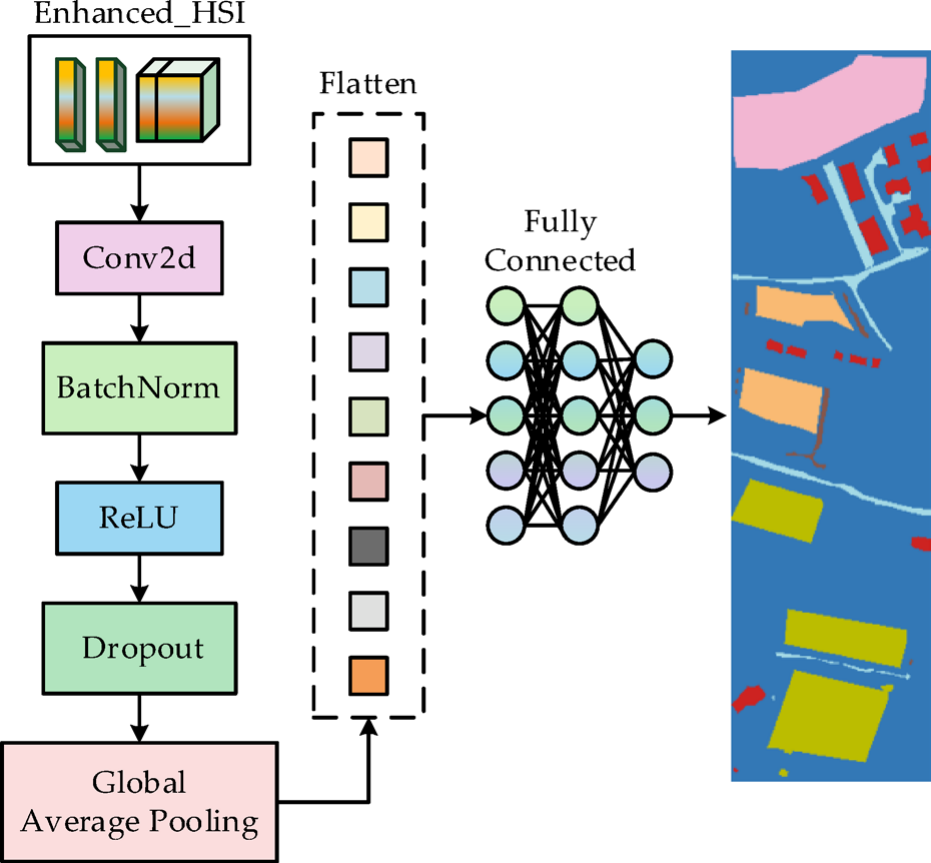
The structure diagram of SSR-Classifier.

After completing feature enhancement through the CPAF module and L-GNIF Unit, the resulting enhanced hyperspectral features $\left( {enhanced\_hsi} \right)$ form an information dense, high-dimensional spatial-spectral feature map. Although of high generation quality, it remains essentially a complex feature representation that cannot be directly used for final classification decisions. To address this mapping challenge from “high-level features” to “specific categories”, we propose the spatial-spectral refinement classifier (SSR-Classifier) as the network’s efficient “decision terminal”, with its structure shown in Fig. 5.

The SSR-Classifier represents a computationally efficient CNN architecture optimized for hyperspectral image classification applications. Its front-end employs 3 × 3 convolutional layers to perform deep spatial-spectral feature refinement on the fused enhanced hyperspectral features, aiming to compress multi-band information into higher-level feature channels and achieve joint local spatial-spectral encoding, thereby effectively extracting low-order local correlation features. Subsequently, in the feature transformation pipeline, BatchNorm and ReLU activation functions form a “stability enhancement unit”. The former accelerates training convergence and mitigates gradient vanishing through standardization, while the latter introduces nonlinearity to break linear mapping limitations, synergistically improving the robustness of feature representation. Furthermore, to further compress redundancy and achieve global feature modeling, the network’s tail incorporates a combined mechanism of global average pooling and adaptive average pooling, balancing the capture of fixed-scale and variable-scale information to generate a 1 × 1 × C embedding vector. Through a fully-connected layer operation, the vector is converted to class probability scores that form the model’s output predictions.

### PSR-discriminator

In the GAN architecture, the primary objective of the discriminator is to evaluate the discrepancy between generated samples and real samples, thereby guiding the feature learning of the generator. However, conventional discriminators typically rely solely on a binary real/fake label as the feedback signal, which is often insufficient for fully capturing the complex spectral-spatial hierarchical relationships inherent in hyperspectral imagery.

**Fig. 6:**
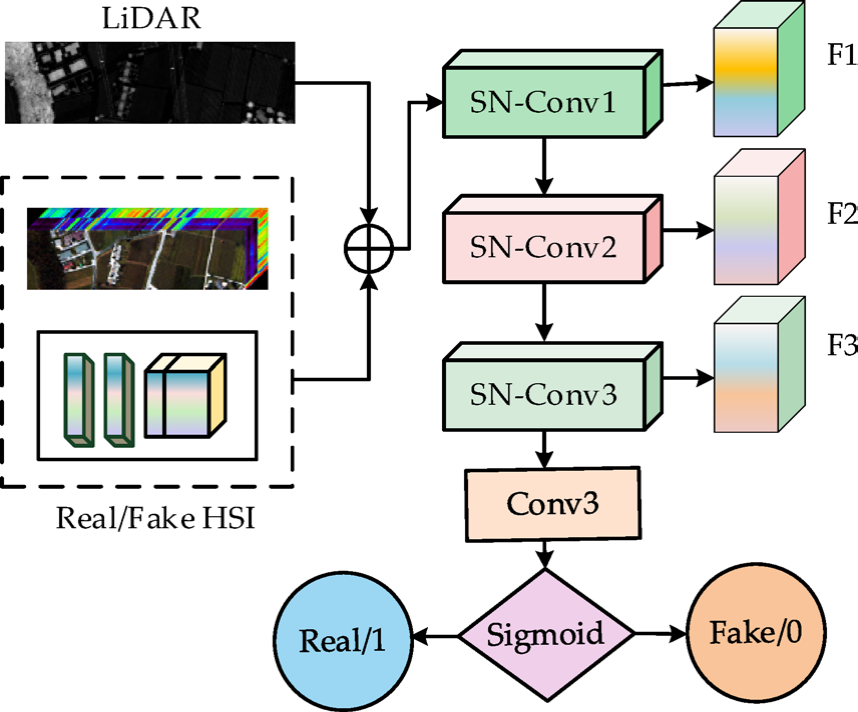
The structure diagram of PSR-Discriminator.

To address this limitation, we designed a perception-enhanced spectral regularization discriminator (PSR-Discriminator), whose structure is illustrated in Fig. 6. This module employs a multi-scale convolutional architecture (SN-Conv1 ~ 3) to extract progressively deeper spectral-spatial features from the input image (comprising concatenated LiDAR data and either real HSI data or HSI data generated by the generator). Spectral normalization^25^ is applied to each convolutional layer to stabilize the training process and prevent mode collapse. Unlike traditional binary classifiers, the PSR-Discriminator not only outputs a real/fake discrimination but also retains the intermediate feature maps (F1 ~ F3) from each convolutional stage, thereby providing more granular structural information for generator training.

During training, these intermediate feature maps (from SN-Conv1 ~ 3) are utilized to compute a feature matching constraint: when both real and generated images are processed by the discriminator, the generator is driven to minimize the differences between their respective representations in the corresponding feature spaces. Through this mechanism, the generator learns not only to replicate pixel-level statistical similarities with real samples but also to approximate the true data distribution at the level of high-level semantics and spatial structure. Since the SSR-Classifier is directly embedded at the output of the generator, this feature matching constraint indirectly enhances the spectral consistency and semantic discriminability of the generated images through backpropagation. Consequently, it provides the classifier with higher-quality input features, significantly boosting overall classification performance and generalization capability.

### Joint multi-task optimization loss function

To jointly optimize the entire framework, we designed a joint multi-task optimization loss function $L_{total}$ consisting of generator loss $L_{G}$ and discriminator loss $L_{D}$:

7 $$L_{total} = L_{G} + L_{D}$$

The discriminator loss $L_{D}$ adopts the standard adversarial loss formulation, aiming to maximize its ability to distinguish real and fake samples:

8 $$L_{D} = - {\mathbb{E}}_{{(x_{H} ,x_{L} )\sim P_{{{\text{data}}}} }} [\log D(x_{H} ,x_{L} )] - {\mathbb{E}}_{{x_{H} ,x_{L} }} [\log (1 - D(G(x_{H} ,x_{L} ),x_{L} ))]$$

The generator’s total loss $L_{G}$ is a multi-task loss weighted by three components-classification loss, adversarial loss, and feature matching loss-to achieve balanced optimization of classification accuracy, feature authenticity, and perceptual similarity:

9 $$L_{G} = \lambda_{cls} L_{cls} + \lambda_{adv} L_{adv} + \lambda_{feat} L_{feat}$$

The balancing hyperparameters $\lambda_{cls} ,\lambda_{adv} ,\lambda_{feat}$ are manually set as fixed scalars. Their values were determined through a series of grid-search experiments on the validation set to ensure that all three loss components contribute commensurately to the final performance. In all experiments of this study, the final adopted values are:$\lambda_{cls} = 25.0,\lambda_{adv} = 0.5,\lambda_{feat} = 8.0$. The relatively high value of $\lambda_{cls}$ emphasizes that high classification accuracy is our primary goal, while $\lambda_{adv}$ and $\lambda_{feat}$ are used to stabilize the adversarial training and preserve the structural fidelity of the generated features, respectively.

The specific definitions of each loss component are as follows:

1. Classification Loss $L_{cls}$: we employ focal loss instead of cross-entropy to better handle class imbalance by emphasizing hard-to-classify samples.

(2) Adversarial Loss $L_{adv}$: this loss enhances the authenticity of generated features by encouraging the discriminator to assign higher “real” scores to generated samples, defined as:
  10 $$L_{adv} = - {\mathbb{E}}_{{x_{H} ,x_{L} }} [\log D(G(x_{H} ,x_{L} ),x_{L} )]$$

where,$x_{H} ,x_{L}$ represents the hyperspectral and LiDAR data, and ${\mathbb{E}}$ denotes the expected value.

(3) Feature Matching Loss $L_{feat}$: to further improve generation quality, we add feature matching loss that measures L1 distance between real and synthetic sample features. This guides the generator to learn more meaningful representations:
  11 $$L_{feat} = \sum\limits_{i = 1}^{k} {\frac{1}{{N_{i} }}} ||D_{i} (x_{H} ,x_{L} ) - D_{i} (G(x_{H} ,x_{L} ),x_{L} )||_{1}$$

where,$D_{i}$ represents the output of the i-th intermediate layer in the discriminator, $k$ is the number of selected layers, and $\lambda_{cls} ,\lambda_{adv} ,\lambda_{feat}$ are hyperparameters balancing different loss terms.

## Experimental results and analysis

This section conducts experimental verification on the performance of the ELDGG algorithm in the fusion and classification of HSI and LiDAR data, covering experimental datasets, parameter settings, evaluation metrics, ablation experiments, and comparative experiments, etc. The details are as follows:

### Datasets

This study employs three public datasets, Houston2013, Trento and MUUFL, as benchmarks for performance evaluation. For each dataset, the land cover classification scheme and the partitioning of samples into training and test sets are explicitly shown in Tables 1, 2, and 3. It should be noted that due to inherent class imbalance within the dataset, the stratified sampling strategy adopted in this study may result in the number of training samples for certain rare categories (e.g., ‘Water’ in Table 1) being comparable to or even slightly exceeding their corresponding test set quantities. This approach was implemented specifically to ensure adequate representation of minority classes during the training process.

**Table 1:** Houston2013 dataset.

**Class**	**Class name**	**Train**	**Test**	**Class**	**Class name**	**Train**	**Test**
C1	Health grass	198	1053	C9	Road	193	1059
C2	Stressed grass	190	1064	C10	Highway	191	1036
C3	Synthetic grass	192	505	C11	Railway	181	1054
C4	Trees	188	1056	C12	Parking lot 1	192	1041
C5	Soil	186	1056	C13	Parking lot 2	184	285
C6	Water	182	143	C14	Railway	181	247
C7	Residential	196	1072	C15	Running track	187	473
C8	Commercial	191	1053		Total	2832	12,197

**Table 2:** Trento dataset.

**Class**	**Class name**	**Train**	**Test**	**Class**	**Class name**	**Train**	**Test**
C1	Health grass	198	1053	C4	Woods	154	9123
C2	Stressed grass	190	1064	C5	Vineyard	184	10,501
C3	Synthetic grass	192	505	C6	Roads	122	3174
					Total	819	30,214

**Table 3:** MUUFL dataset.

**Class**	**Class name**	**Train**	**Test**	**Class**	**Class name**	**Train**	**Test**
C1	Trees	200	23,046	C7	Building shadow	200	2033
C2	Mostly grass	200	4070	C8	Building	200	6040
C3	Mixed ground surface	200	6682	C9	Sidewalk	138	1247
C4	Dirt and sand	182	1644	C10	Yellow curb1	18	165
C5	Road	200	6487	C11	Cloth panels	26	243
C6	Water	46	420		Total	1610	52,077

### Experimental setup

1. Subjective and objective assessment indicators:

This study adopts a comprehensive evaluation strategy combining quantitative metrics and visual analysis. Quantitative assessment includes four widely-used measures: Overall Accuracy (OA), Average Accuracy (AA), Kappa Coefficient (Kappa), and per-class accuracy. Qualitative evaluation examines classification maps through com-parison with ground truth and error distribution maps, focusing on four key aspects: regional homogeneity, boundary clarity, small-object completeness, and error distribution patterns.

(2) Experimental Configuration:

All experiments were implemented using PyTorch on workstations equipped with NVIDIA RTX 3090 GPUs. To ensure fair comparison: baseline methods were reproduced using official code, with missing details supplemented according to original papers; fixed random seeds were used for data splitting (10% training for DL methods, 30% for traditional methods); all methods employed their originally reported optimal hyperparameters; reported results represent averages from five independent runs.

### Analysis of hyperparameter effects

**Fig. 7:**
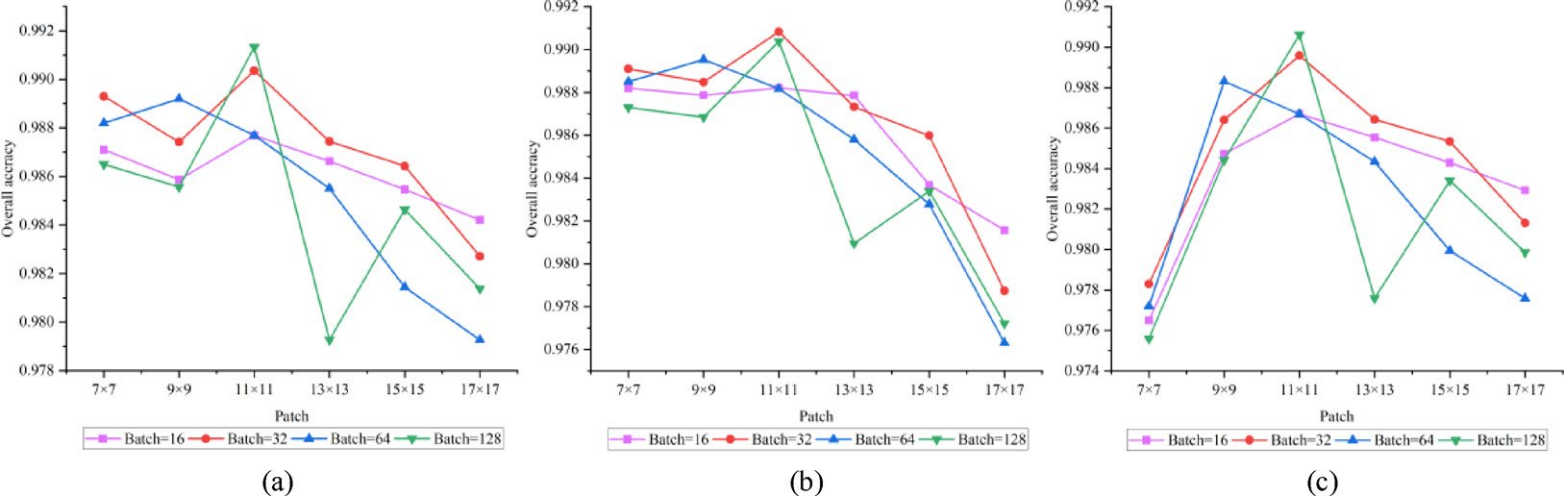
Houston2013 dataset. (**a**)OA;(**b**) AA;(**c**) Kappa.

**Fig. 8:**
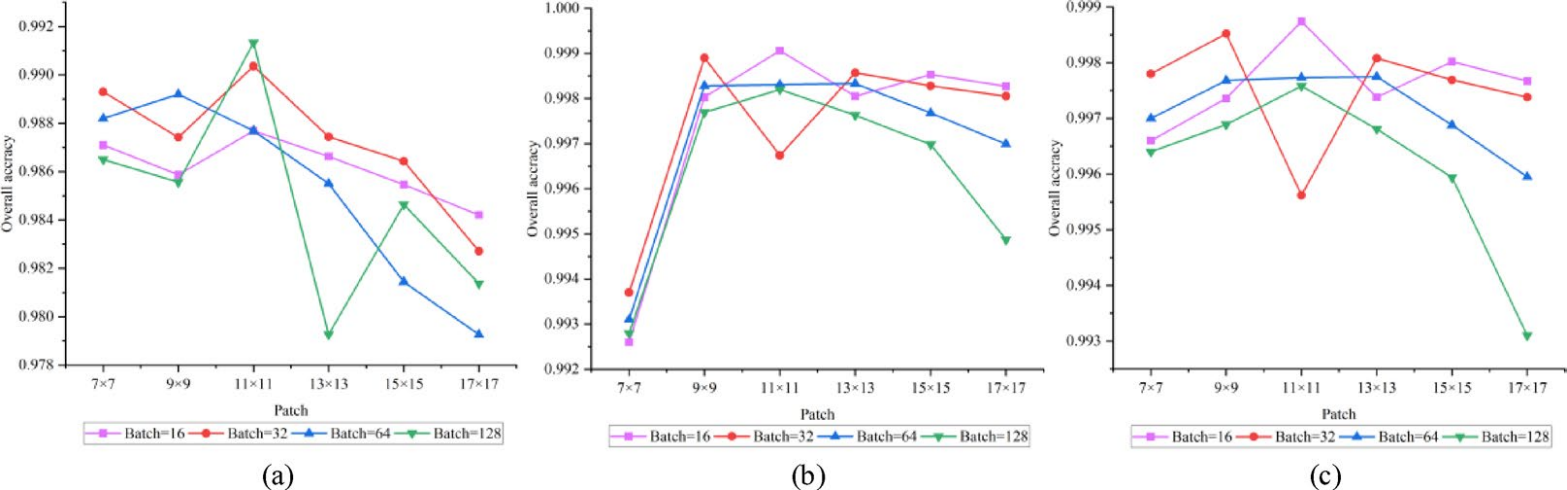
Trento dataset. (**a**)OA;(**b**) AA;(**c**) Kappa.

**Fig. 9:**
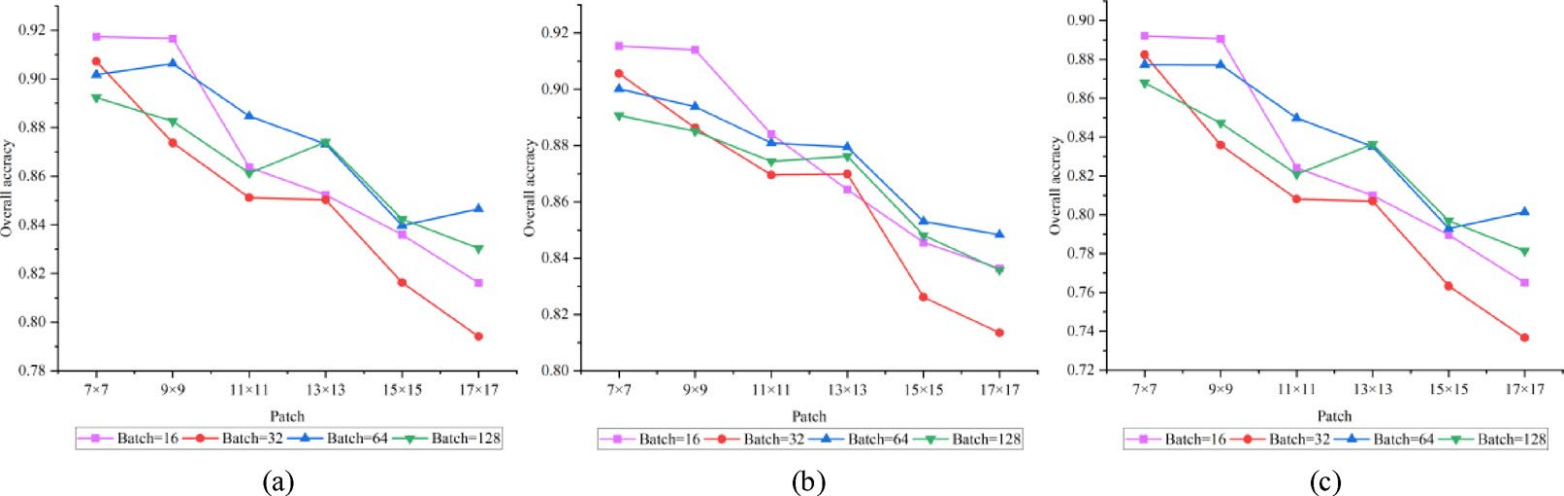
MUUFL dataset. (**a**)OA;(**b**) AA;(**c**) Kappa.

In evaluating the impact of critical hyperparameters on model efficacy, we examined two factors: patch size (Patch) and batch size (Batch) . In the experiments, we tested six different patch sizes ranging from 7 × 7 to 17 × 17, as well as four batch sizes (16, 32, 64, and 128). The experimental results for each parameter combination across three datasets are illustrated in the accompanying Figs. 7, 8, 9.

The results reveal notable variations in optimal parameter configurations across different datasets, reflecting the influence of spatial structure, class distribution, and other inherent characteristics of remote sensing data on model performance. On the Houston2013 dataset, the model achieved its highest OA of approximately 0.9915 with a patch size of 11 × 11 and a batch size of 128. This suggests that for complex urban scenes with mixed land cover, a moderately large receptive field helps capture richer contextual information, thereby improving classification performance. In contrast, the MUUFL dataset performed best with a smaller patch size (7 × 7) and a smaller batch size (16), achieving an OA of 0.9180. This trend may be attributed to the dataset’s fragmented feature regions and dense small targets, where larger patch sizes could introduce heterogeneous interference. On the Trento dataset, the optimal configuration was a 9 × 9 patch size and a batch size of 16, yielding an OA close to 0.9990. This combination balances receptive field coverage while mitigating over-smoothing risks, effectively adapting to the dataset’s structured farmland and building features.

Overall, model performance is sensitive to both patch size and batch size, with optimal settings varying across datasets depending on spatial resolution, object distribution, and class complexity. These findings provide valuable insights for parameter selection in similar tasks.

Furthermore, to evaluate the practical deploy ability of the model, we computed computational complexity metrics on the Houston2013 dataset (Patch = 11 × 11, Batch = 128). The results are as follows: the total number of model parameters is approximately 5.76M, the average training time per epoch is 5.23 s (total training duration is approximately 0.13 h), and the average inference time is 5.10 ms/sample. These performance indicators demonstrate that although the proposed hierarchical reconstruction architecture introduces multi-level L-GNIF Units, through channel compression and lightweight MLP design (the parameter generator has only 4.26M parameters), the model maintains high accuracy while achieving high computational efficiency. This enables it to meet the real-time and resource constraints required in practical remote sensing applications.

### Ablation experiment

1. Component-wise evaluation of individual data modalities:

To evaluate the actual contribution of multimodal data fusion in the model and investigate the respective impacts of HSI and LiDAR data on classification performance, we designed comparative ablation experiments. Specifically, we trained and tested the model using single-modal data (Only HIS or Only LiDAR) and compared the results with the complete dual-modal fusion model (ELDGG). The results are summarized in Table 4.

**Table 4:** Component-wise evaluation of individual data modalities.

**Experiment**	**Houston2013**			**Trento**			**MUUFL**		
	OA	AA	Kappa	OA	AA	Kappa	OA	AA	Kappa
Only HSI	0.9762	0.9765	0.9751	0.9585	0.9315	0.9442	0.8235	0.8339	0.7959
Only LiDAR	0.6497	0.6683	0.6237	0.8281	0.6262	0.7574	0.4922	0.5147	0.4775
ELDGG	**0.9859**	**0.9879**	**0.9847**	**0.9980**	**09968**	**0.9974**	**0.9116**	**0.9141**	**0.8906**

Bold denotes optimal values.

The experimental results demonstrate that in complex urban remote sensing scenarios with ambiguous class boundaries, such as Houston2013 and MUUFL, the fusion model significantly outperforms single-modal inputs across all evaluation metrics, including OA, AA, and Kappa. This highlights the effectiveness of multisource data fusion for complex land cover recognition. Particularly on the MUUFL dataset, ELDGG achieves an OA of 0.9116, representing an approximately 9% improvement over HSI-only data (0.8235) and a remarkable 40% enhancement compared to LiDAR-only data (0.4922). These results indicate strong complementarity between the two modalities: HSI provides rich spectral details, while LiDAR supplements spatial structure and elevation features, with their synergistic effects being crucial for improving classification robustness.

Notably, on the Trento dataset, the classification accuracy using HSI-only data slightly surpasses that of the fusion model. We hypothesize that this may be attributed to the relatively limited information gain from LiDAR data in this dataset, where the introduced spatial features might even cause minor interference with spectral discrimination in certain regions. Nevertheless, considering the overall trends across all three datasets, multimodal fusion consistently demonstrates more robust performance improvements, particularly in scenarios with stronger heterogeneity.

(2) Ablation analysis of different components

To systematically evaluate the effectiveness of core components in the ELDGG framework, we conducted rigorous ablation experiments on the Houston2013 dataset. The experimental setup was as follows: Baseline model E1 adopted traditional up sampling and simple feature concatenation without any innovative components, E2 removed the GAN adversarial training mechanism, E3 excluded the CPAF-Module, E4 disabled the L-GNIF Unit, and E5 represented the complete model. All experiments maintained identical hyperparameter settings and training strategies, with results quantitatively analyzing each component’s specific contribution to model performance.

As clearly shown in Table 5, our complete model (E5) achieved the best performance across all three comprehensive metrics. The baseline model (E1) exhibited the lowest performance, while the absence of any single innovative component (E2, E3, E4) resulted in intermediate performance between the baseline and complete model. This preliminarily confirms that each designed module positively contributes to the final performance. Comparing E5 and E2, removing the adversarial learning mechanism led to significant declines of 0.0268 in OA, 0.0242 in AA, and 0.0289 in Kappa, the most pronounced performance drops among all single-module ablations. This underscores adversarial training’s indispensable role as a powerful regularization method for enhancing feature authenticity and model generalization. The comparison between E5 and E3 revealed a 0.0146 OA reduction when replacing the parameter-adaptive fusion module with simple feature concatenation. This demonstrates that the CPAF Module, through sample-level dynamic parameter generation, enables more effective cross-modal interaction than static concatenation, establishing it as one of the core technologies for performance improvement. Lastly, E5 versus E4 showed a 0.0093 OA decrease upon removing the high-fidelity reconstruction unit. This validates the effectiveness of our neural implicit representation-based feature reconstruction. By generating smoother feature maps with sharper boundaries, the module provides higher-quality inputs to the classifier, further optimizing performance.

**Table 5:** Performance metrics of individual modules evaluated on the Houston2013 dataset.

Experiment	GAN	CPAF-Module	L-GNIF Unit	OA	AA	Kappa
E1	–	–	–	0.9515	0.9554	0.9475
E2	–	√	√	0.9591	0.9637	0.9558
E3	√	–	√	0.9713	0.9743	0.9690
E4	√	√	–	0.9766	0.9770	0.9747
E5	√	√	√	**0.9859**	**0.9879**	**0.9847**

Bold denotes optimal values.

**Fig. 10:**
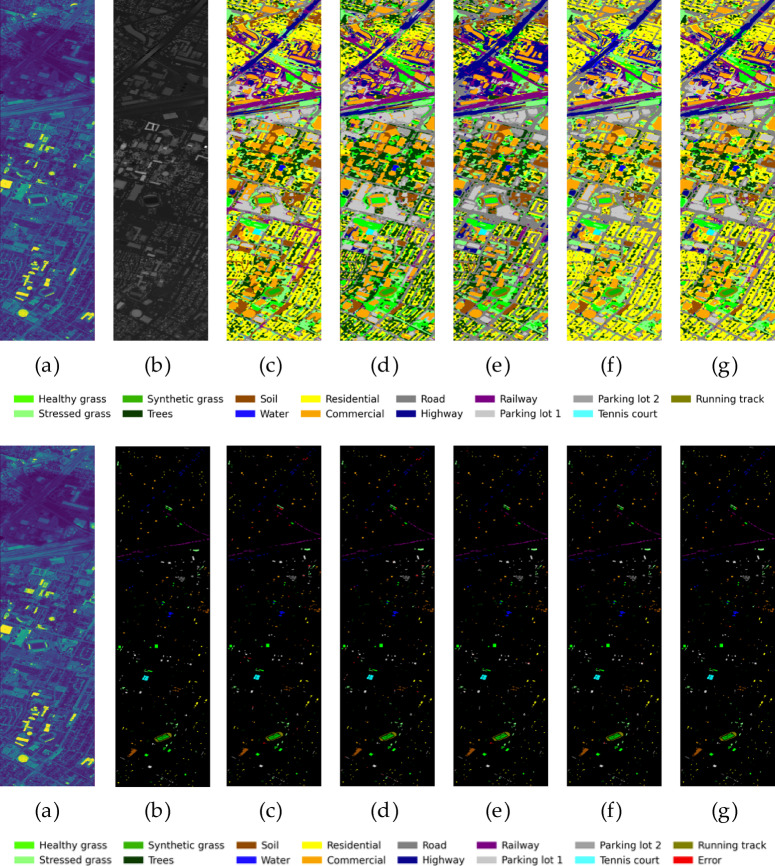
Classification performance and error distribution plots for various modules evaluated on the Houston2013 dataset. From left to right: (**a**) HSI, (**b**) LiDAR, (**c**)E1, (**d**) E2, (**e**) E3, (**f**) E4, (**g**) E5.

To more intuitively evaluate the functions of each module, Fig. 10 presents the classification result graphs of each core component and their corresponding error graphs. A clear process of “picture quality” improvement can be discovered through visual comparison. The classification map of the benchmark model (c) shows the most obvious “salt and pepper noise”, with fragmented plots and blurred boundaries. After the introduction of CPAF-Module and L-GNIF Unit, the smoothness and regional integrity of the image have been improved to a certain extent, but there are still many mis-divided patches. After further incorporating adversarial training, the Outlines of ground objects, especially linear ones like roads, began to become clearer. Ultimately, our complete model (g) performed best in terms of visual effects. The classification map it generates has the cleanest overall appearance. There are almost no color spots inside large homogeneous areas (such as residential areas), and the boundaries of various ground features are also the sharpest and most natural. By observing the error graph in the second row, it can be found that from (d) to (g), the number of misaligned pixels highlighted in red shows a decreasing trend step by step, which is consistent with the result of quantitative analysis.

In conclusion, through the dual verification of quantitative indicators and qualitative visual effects, the GAN framework, CPAF-Module, and L-GNIF Unit designed in this study have all played a crucial and quantifiable positive role in improving the final classification performance, proving the rationality and advancement of our overall framework design.

### Comparative experiment

To rigorously validate the efficacy of the proposed approach, we conducted comparative experiments on two standard datasets, Houston2013 and Trento, and selected seven representative methods for comparative analysis, including the machine learning method SVM and six deep learning methods: FusAtNet^26^, CCRNet^27^, MAHiDFNet^16^, GLT^28^, ExViT^29^and MFT^30^. These comparison methods cover the main technical routes of current multimodal remote sensing image classification: SVM, as a classic kernel method, needs to rely on artificially designed spatial-spectral features for classification; FusAtNet uses a self-attention mechanism to generate attention maps of hyperspectral images and employs LiDAR data for cross-attention enhancement; CCRNet is based on the encoder-decoder framework and achieves cross-modal feature extraction through triple convolutional blocks; MAHiDFNet innovatively constructed a three-branch CNN structure, extracting spatial, spectral, and elevation features respectively, and achieving hierarchical fusion through the modal attention module; GLT effectively bridges CNN’s strength in local pattern extraction and Transformer’s capacity for long-range dependency modeling; ExViT performs feature synthesis after processing multimodal data through separable convolution and Transformer encoders, respectively; and MFT employs 3D-2D heterogeneous convolution to process hyperspectral data and utilizes a cross-modal attention mechanism to promote feature integration. Detailed experimental results and comparative analyses will be discussed in the subsequent sections.

Tables 6, 7, and 8 present the quantitative evaluation results of different methods on the Houston2013, MUUFL and Trento datasets, respectively. A consistent trend can be observed across both datasets: compared to traditional machine learning methods like SVM, deep learning-based approaches generally demonstrate superior performance. This indicates that deep learning models possess greater potential in automatically extracting high-level spectral-spatial features from remote sensing data, as opposed to relying on manual feature engineering in conventional methods.

**Table 6:** Performance metrics comparison across methodologies using the Houston2013 dataset.

Class	SVM	FusAtNet	CCRNet	MAHiDFNet	GLT	MFT	ExViT	ELDGG
C1	0.8747	0.9856	0.9796	0.9813	0.9565	0.9710	0.9221	**0.9796**
C2	0.9947	0.9928	0.9646	0.9911	0.9973	0.9930	**0.9980**	0.9575
C3	0.9904	0.9957	0.9984	**1.0000**	**1.0000**	0.9964	0.9946	0.9968
C4	0.9866	0.9871	0.9973	**1.0000**	0.9982	0.9799	**1.0000**	0.9991
C5	0.9973	0.9847	**1.0000**	**1.0000**	**1.0000**	0.9990	**1.0000**	**1.0000**
C6	0.8469	0.7754	**1.0000**	0.9966	0.8191	0.8736	0.9923	0.9966
C7	0.8816	0.9890	0.9851	0.9842	0.9799	0.9734	**1.0000**	**1.0000**
C8	0.8606	0.9598	**0.9670**	0.9098	0.9982	0.9016	0.9197	0.9434
C9	0.8112	0.9832	0.9414	**0.9911**	0.9397	0.8433	0.9631	0.9885
C10	0.8315	0.9658	0.9258	0.8234	0.9937	0.7721	**0.9949**	0.9810
C11	0.8356	0.9652	0.9658	0.9317	1.0000	0.9899	0.9777	**0.9982**
C12	0.8108	0.9416	0.9649	0.9883	0.9486	0.9686	0.9838	**0.9892**
C13	0.3759	0.7377	0.9594	0.9716	0.9551	0.8966	0.9521	**0.9882**
C14	0.9766	0.9089	**1.0000**	**1.0000**	**1.0000**	**1.0000**	**1.0000**	**1.0000**
C15	0.9899	0.9924	**1.0000**	**1.0000**	**1.0000**	**1.0000**	**1.0000**	**1.0000**
OA	0.8833	0.9636	0.9731	0.9662	0.9791	0.9437	0.9781	**0.9859**
AA	0.8710	0.9443	0.9766	0.9713	0.9724	0.9439	0.9799	**0.9879**
Kappa	0.8737	0.9606	0.9709	0.9635	0.9774	0.9391	0.9764	**0.9847**

Bold denotes optimal values.

**Table 7:** Performance metrics comparison across methodologies using the Trento dataset.

Class	SVM	FusAtNet	CCRNet	MAHiDFNet	GLT	MFT	ExViT	ELDGG
C1	0.8860	0.9990	0.9948	0.9893	**1.0000**	0.9947	0.9957	0.9974
C2	0.9724	0.9883	0.9923	**1.0000**	0.9778	0.9458	0.9724	0.9943
C3	0.9583	0.7599	0.9674	0.9698	0.5648	0.8494	0.7370	**1.0000**
C4	0.9985	**1.0000**	**1.0000**	**1.0000**	**1.0000**	0.9999	**1.0000**	**1.0000**
C5	0.9432	0.9823	0.9998	**1.0000**	**1.0000**	**1.0000**	0.9949	**1.0000**
C6	0.9569	0.9272	0.8663	0.8466	**0.9982**	0.9193	0.9961	0.9881
OA	0.9568	0.9811	0.9839	0.9820	0.9908	0.9832	0.9904	**0.9979**
AA	0.9526	0.9428	0.9701	0.9676	0.9235	0.9515	0.9493	**0.9966**
Kappa	0.9423	0.9748	0.9786	0.9760	0.9877	0.9775	0.9872	**0.9972**

Bold denotes optimal values.

**Table 8:** Performance metrics comparison across methodologies using the MUUFL dataset.

Class	SVM	FusAtNet	CCRNet	MAHiDFNet	GLT	MFT	ExViT	ELDGG
C1	0.9675	**0.9946**	0.9572	0.9679	0.9840	0.9573	0.9474	0.9178
C2	0.7440	0.1717	0.7937	0.3445	0.8270	0.8762	0.7509	**0.8821**
C3	0.8334	**0.9914**	0.8100	0.9889	0.8907	0.8885	0.9655	0.8602
C4	0.8777	0.8762	0.9526	0.8649	0.9720	0.9391	**0.9740**	0.9720
C5	0.9482	0.9716	0.9520	0.9619	**0.9694**	0.9387	0.9075	0.9299
C6	0.8000	0.8798	0.9639	0.9809	0.0286	0.9385	**0.9920**	**0.9833**
C7	0.7045	**0.9901**	0.9075	0.9522	0.9055	0.9155	0.8495	0.9636
C8	0.9434	**0.9881**	0.9765	0.9635	0.9856	0.9545	0.9543	0.9624
C9	0.5012	**0.9170**	0.6079	0.8741	0.2799	0.3436	0.3186	0.8540
C10	0.5818	0.6721	0.0265	0.7818	0.0000	0.0000	0.0476	**0.7333**
C11	0.8889	0.8885	0.9909	**0.9959**	0.0000	0.8571	0.9074	**0.9959**
OA	0.8981	0.9163	0.6922	0.9128	**0.9195**	0.9172	0.9076	0.9166
AA	0.7991	0.8492	0.8126	0.8797	0.6621	0.7826	0.7831	**0.9141**
Kappa	0.8646	0.8889	0.6275	0.8850	**0.8934**	0.8910	0.8787	0.8906

Bold denotes optimal values.

As clearly shown in Table 6, our proposed ELDGG achieves the best performance across all three key metrics-OA (0.9859), AA (0.9879), and Kappa (0.9847). Compared to state-of-the-art Transformer-based models like ExViT and GLT, ELDGG improves OA by approximately 0.0078 and 0.0068, demonstrating competitive advantages. These results preliminarily suggest that our generative adversarial framework plays a positive role in feature enhancement and fusion. Notably, our method excels in classifying challenging categories. For instance, it achieves 0.9882 accuracy for C13 (parking Lot 2), a structurally complex and underrepresented class, significantly outperforming other comparative methods. Similarly, it shows higher classification accuracy for spectrally similar categories like C8 (commercial Area) and C9 (road), further validating the model’s ability to enhance fine-grained recognition of complex land covers through high-fidelity reconstruction and adaptive fusion.

As shown in Table 7, the experimental results on the Trento dataset further confirm the effectiveness of our method. ELDGG again achieves leading performance with an OA of 0.9979, AA of 0.9966, and Kappa of 0.9972. Although the Trento dataset features relatively simpler land covers, where many advanced methods already achieve OA above 0.99, our method still delivers marginal yet consistent improvements. This suggests that even in less challenging scenarios, our model’s strong feature representation capability can uncover subtle optimizable details. Additionally, our method achieves perfect 1.0000 accuracy for categories C3, C4, and C5, demonstrating its stability and robustness across different scenarios.

To more comprehensively evaluate the model’s adaptability, we conducted additional experiments on the MUUFL dataset. This dataset is characterized by a large number of classes and extremely imbalanced sample distribution, posing higher demands on model robustness. As shown in Table 8, ELDGG achieves comparable performance to methods like GLT in terms of OA (0.9166) and Kappa (0.8906). However, it attains the highest AA score of 0.9141, outperforming all comparative methods in terms of overall balanced per-formance. It should be noted that while some models demonstrate strong overall accuracy, their high performance on the majority classes often comes at the expense of minority class recognition. For instance, although GLT achieves the highest OA, its AA is only 0.6621—a common “biased learning” phenomenon in datasets with extreme class imbalance. In contrast, ELDGG maintains robust performance across multiple minority classes through its adaptive modeling and fusion of multimodal information. Specifically, it achieves high classification accuracy for classes such as C2 (grass), C6 (water), and C10 (yellow curb), demonstrating its enhanced anti-interference capability when handling class imbalance issues.

**Fig. 11:**
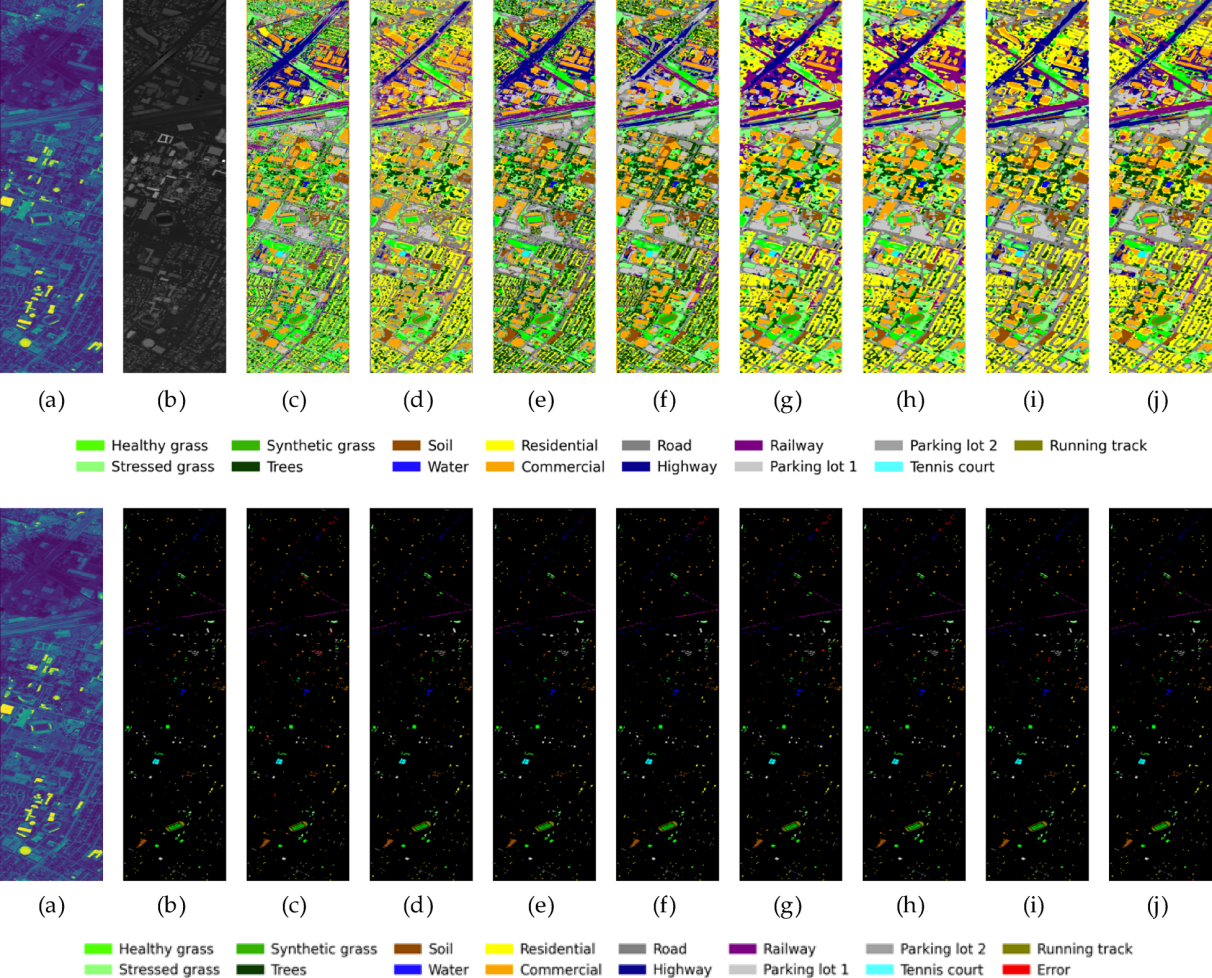
The classification result graphs of different methods on the Houston2013 dataset and their cor-responding error graphs. From left to right: (a) HSI, (**b**) LiDAR, (**c**)SVM, (**d**) FusAtNet, (**e**) CCRNet, (**f**) MAHiDFNet, (**g**) GLT, (**h**) MFT, (**i**) ExViT, (**j**) ELDGG.

**Fig. 12:**
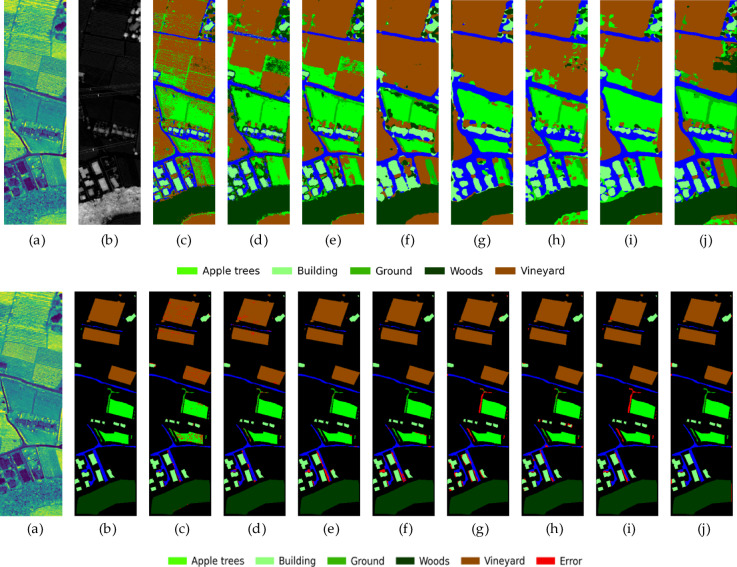
The classification result graphs of different methods on the Trento dataset and their corresponding error graphs. From left to right: (**a**) HSI, (**b**) LiDAR, (**c**)SVM, (**d**) FusAtNet, (**e**) CCRNet, (**f**) MAHiDFNet, (**g**) GLT, (**h**) MFT, (**i**) ExViT, (**j**) ELDGG.

**Fig. 13:**
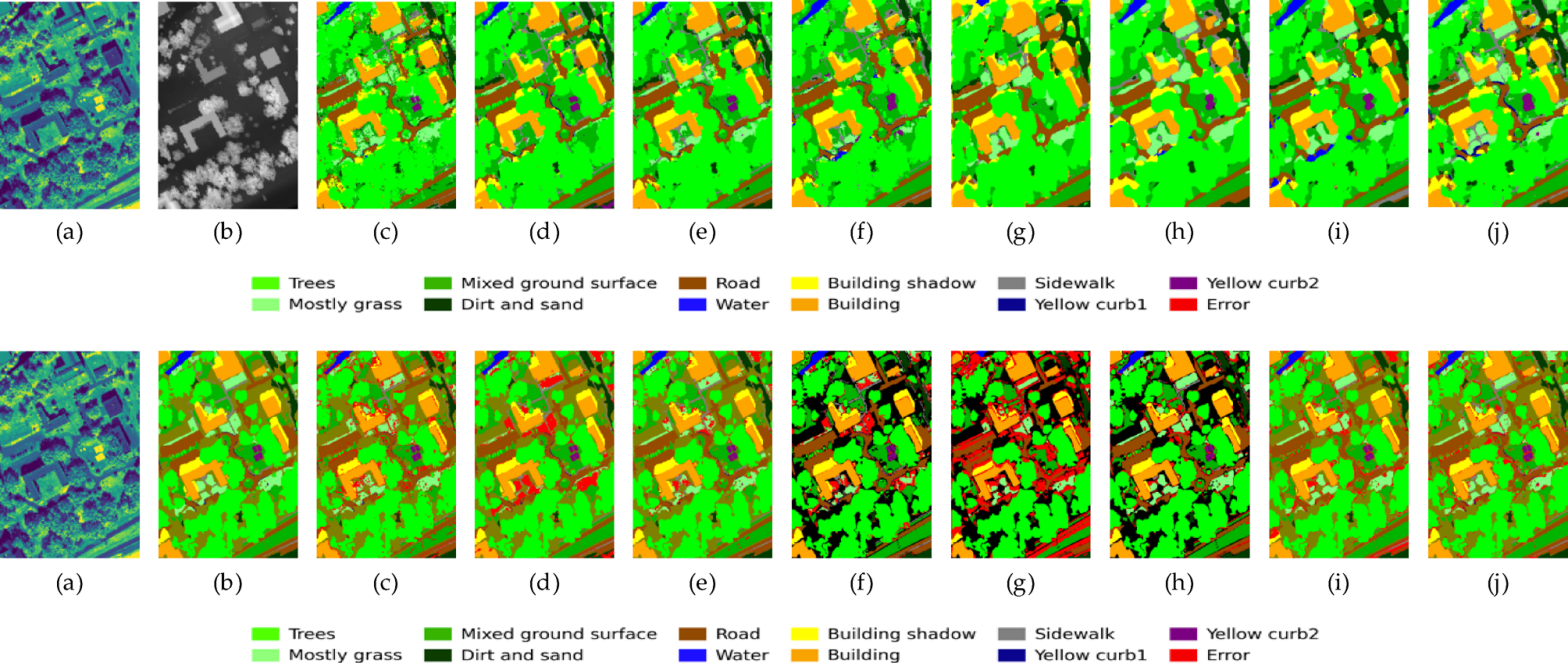
The classification result graphs of different methods on the MUUFL dataset and their corresponding error graphs. From left to right: (**a**) HSI, (**b**) LiDAR, (**c**)SVM, (**d**) FusAtNet, (**e**) CCRNet, (**f**) MAHiDFNet, (**g**) GLT, (**h**) MFT, (**i**) ExViT, (**j**) ELDGG.

To provide a more intuitive assessment of each model’s classification performance, Figs. 11, 12, and 13 present the classification maps and corresponding error maps (with smisclassified pixels highlighted in red) of different methods on the Houston2013 and Trento datasets, respectively. A comparison of the error maps reveals the relative strengths and weaknesses of each algorithm more clearly.

For the Houston2013 dataset (Fig. 11), our method produces classification results with significantly improved visual smoothness and regional homogeneity. Large homogeneous areas, such as C7 (residential) and C5 (soil), exhibit notably reduced “salt-and-pepper” noise while maintaining better spatial consistency. In contrast, SVM yields fragmented classification patches, while methods like MFT and GLT still display scattered misclassifications. Our approach also demonstrates superior performance in delineating linear features such as roads (gray) and highways (dark blue), with enhanced continuity and boundary clarity-likely attributable to the high-fidelity spatial detail reconstruction enabled by the L-GNIF Unit. The error maps (second row) further highlight our method’s advantages: ELDGG shows the sparsest and most dispersed distribution of red error pixels among all compared methods, whereas errors in other approaches tend to cluster around class boundaries. This suggests our model’s stronger capability in handling ambiguous edge regions.

On the Trento dataset (Fig. 12), our method’s refined classification ability is equally evident. For agricultural fields (C5 vineyard, brown; C6 ground, green) and forested areas (C4 woods, dark green), our results achieve more precise boundary segmentation, with regularized parcel shapes that closely match the actual terrain. Other methods, however, exhibit varying degrees of “mutual erosion” between adjacent parcels. Notably, for buildings (C2, light green), our method accurately outlines their geometric structures, with virtually no building-related errors in the corresponding error map. This again underscores the importance of LiDAR-guided high-fidelity reconstruction in preserving the geometric integrity of artificial features.

To further validate the model’s generalization capability in more complex urban remote sensing scenarios, we conducted visual analysis on the MUUFL dataset (Fig. 13). From a holistic perspective, the results generated by our proposed method demonstrate superior performance in spatial consistency and regional smoothness, significantly reducing noise-induced fragmented areas while maintaining coherent land-cover structures. Specifically, the contours of large building clusters in the central area are well preserved, with our method clearly distinguishing building bodies (yellow), shadows (dark blue), and surrounding vegetation (green). In contrast, other methods (e.g., MFT, ExViT) exhibit varying degrees of class confusion at boundary regions, resulting in dense red pixel bands in the error maps. Moreover, our method demonstrates strong capability in preserving small targets (e.g., roads, sidewalks) without being “overwhelmed” by surrounding areas. The error map reveals that misclassifications are primarily concentrated at transitional boundaries between classes, with overall errors being relatively sparse and scattered. Comparatively, some methods (e.g., GLT) produce large patchy misclassifications, indicating limitations in their feature modeling approaches.

In summary, whether applied to highly complex scenarios with cloud/fog occlusion and diverse land-cover types (Houston dataset), simpler scenes with limited land-cover categories (Trento dataset), or challenging urban environments with densely distributed small targets and class imbalance (MUUFL dataset), our proposed ELDGG framework consistently demonstrates competitive classification performance and robust generalization capability. This is achieved through its unique adaptive fusion mechanism, high-fidelity reconstruction, and adversarial learning strategy.

## Conclusions

To address the challenges of insufficient cross-modal information interaction and distortion-prone feature reconstruction in deep networks for hyperspectral and LiDAR data fusion classification tasks, this paper proposes the ELDGG. This framework organically integrates adaptive fusion, neural implicit reconstruction, and adversarial learning mechanisms to generate more authentic and discriminative fused features, thereby improving classification accuracy. First, the CPAF-Module addresses the limitations of static fusion by dynamically generating HSI-specific convolutional kernels using LiDAR’s global elevation information, achieving parameter-level information interaction. Second, the L-GNIF Unit employs continuous coordinate mapping technology to achieve subpixel-level spatial detail reconstruction while preserving spectral features. Finally, the PSR-Discriminator innovatively combines spectral constraints with multi-scale feature output supervision, ensuring the physical authenticity of generated features across texture, structure, and semantic levels. Additionally, the entire network adopts an end-to-end joint optimization strategy. By minimizing the multi-task loss function, it not only generates highly discriminative intermediate feature representations but also achieves accurate final classification through SSR-Classifier. Experiments on public datasets such as Houston2013 and Trento demonstrate that the proposed method outperforms multiple comparative algorithms in key metrics including OA, AA, and Kappa. Notably, it exhibits significant advantages in cloud-occluded regions and fine-grained vegetation classification tasks, effectively validating the robustness and inter-class separability of the proposed algorithm. Ablation studies further confirm the positive contributions of each component, including the GAN framework, CPAF-Module, and L-GNIF Unit. Among them, the GAN framework ensures both the physical authenticity of generated features and improved classification accuracy, while the CPAF-Module and L-GNIF Unit positively impact cross-modal feature fusion and spatial detail preservation, respectively.

Future work will focus on three key directions: First, to address the trade-off between overall accuracy and minority class recognition observed in the MUUFL dataset, we plan to investigate more efficient feature interaction mechanisms to enhance the model’s generalization capability under class-imbalanced conditions; second, investigating the framework’s potential in few-shot or semi-supervised learning scenarios to reduce reliance on large amounts of labeled data, and extending the method to fusion tasks with other modalities such as SAR.

## Data Availability

The Houston dataset is available at: http://dase.grss-ieee.org/; the Trento dataset can be obtained at: https://github.com/A-Piece-Of-Maple/TrentoDateset; and the MUUFL dataset is available at: https://github.com/GatorSense/MUUFLGulfport.
